# Left ventricular remodeling and dysfunction in obstructive sleep apnea: Systematic review and meta-analysis

**DOI:** 10.1007/s00059-019-04850-w

**Published:** 2019-09-25

**Authors:** Lei Yu, Huajun Li, Xianbao Liu, Jiaqi Fan, Qifeng Zhu, Jing Li, Jubo Jiang, Jian’an Wang

**Affiliations:** 1 Department of Ultrasonography, The First Affiliated Hospital, Zhejiang University School of Medicine, Hangzhou, China; 2 Department of Cardiology, Second Affiliated Hospital, Zhejiang University School of Medicine, 310009 Hangzhou, China; 3 Zhejiang University School of Medicine, Hangzhou, China

**Keywords:** Left ventricular hypertrophy, Cardiac remodeling, Left ventricular ejection fraction, Hypoxia, Echocardiography, Linksventrikuläre Hypertrophie, Kardiales Remodeling, Linksventrikuläre Ejektionsfraktion, Hypoxie, Echokardiographie

## Abstract

**Background:**

Obstructive sleep apnea syndrome (OSAS) is associated with cardiovascular mortality and morbidity. Several studies have reported that it affects the left ventricle; however, large randomized controlled trials are lacking. The current study aimed to summarize the association between OSAS and left ventricular (LV) structure and function.

**Methods:**

Electronic databases (PubMed, Embase, and Cochrane) and references were searched for articles published until March 2018. A systematic review and meta-analysis were performed to assess LV structure and function in OSAS patients based on echocardiography.

**Results:**

In total, 17 studies with 747 OSAS patients and 426 control participants were included. Patients with OSAS showed an increase in LV diastolic diameter (weighted mean difference [WMD], 95% CI: 1.24 [0.68, 1.80]; *p* < 0.001), LV systolic diameter (WMD, 95% CI: 1.14 [0.47, 1.81]; *p* = 0.001), and LV mass (WMD, 95% CI: 35.34 [20.67, 50.00]; *p* < 0.001). In addition, left ventricular ejection fraction (LVEF) significantly decreased in the OSAS group compared with the controls (WMD, 95% CIs: −1.82 [−2.76, −0.87]; *p* < 0.001), and the reduction in LVEF was consistent with the severity of OSAS. The OSAS group also showed an increase in left atrial diameter (WMD, 95% CI: 2.13 [1.48, 2.77]; *p* < 0.001) and left atrial diameter volume index (WMD, 95% CIs: 3.96 [3.32, 4.61]; *p* < 0.001).

**Conclusion:**

Obstructive sleep apnea syndrome leads to atrial dilatation, left ventricular hypertrophy, enlargement, mass increase and reduction of systolic function. Treatments for OSAS might be beneficial for the preservation of left cardiac structure and function.

**Electronic supplementary material:**

The online version of this article (10.1007/s00059-019-04850-w) contains supplementary material, which is available to authorized users.

## Abstract (de)

**Hintergrund:**

Das obstruktive Schlafapnoesyndrom (OSAS) geht mit kardiovaskulärer Mortalität und Morbidität einher. Im Rahmen verschiedener Studien wurde berichtet, dass es sich auf den linken Ventrikel auswirkt; allerdings fehlen bisher große randomisierte kontrollierte Studien. Die aktuelle Studie zielte darauf ab, eine zusammenfassende Übersicht über den Zusammenhang zwischen OSAS und linksventrikulärer Struktur und Funktion zu geben.

**Methoden:**

Elektronische Datenbanken (PubMed, Embase und Cochrane) sowie Literaturangaben wurden nach Artikeln durchsucht, die bis März 2018 publiziert worden waren. Dann wurden eine systematische Übersicht und eine Metaanalyse erstellt, um die LV-Struktur und Funktion bei Patienten mit OSAS auf der Grundlage der Echokardiographie zu untersuchen.

**Ergebnisse:**

Insgesamt wurden 17 Studien mit 747 OSAS-Patienten und 426 Kontrollen in die Studie einbezogen. Patienten mit OSAS zeigten einen Anstieg des diastolischen Durchmessers des LV (gewichtete mittlere Differenz, WMD: 1,24; 95 %-KI, 95 %-KI: 0,68–1,80; *p* < 0,001), des systolischen Durchmessers des LV (WMD: 1,14; 95 %-KI: 0,47–1,81; *p* = 0,001) und der LV-Masse (WMD: 35,34; 95 %-KI: 20,67–50,00; *p* < 0,001). Außerdem nahm die linksventrikuläre Ejektionsfraktion (LVEF) signifikant in der OSAS-Gruppe im Vergleich zu den Kontrollen ab (WMD: −1,82; 95 %-KI: −2,76 bis −0,87; *p* < 0,001), und die Abnahme der LVEF stand in Übereinstimmung mit dem Schweregrad des OSAS. In der OSAS-Gruppe zeigte sich auch ein Anstieg des linksatrialen Durchmessers (WMD: 2,13; 95 %-KI: 1,48–2,77; *p* < 0,001) und des Volumenindex des linksatrialen Durchmessers (WMD: 3,96; 95 %-KI: 3,32–4,61; *p* < 0,001).

**Schlussfolgerung:**

Ein obstruktives Schlafapnoesyndrom führt zur Vorhofdilatation, linksventrikulären Hypertrophie, Vergrößerung, Massenzunahme und Abnahme der systolischen Funktion. Die Behandlung eines OSAS könnte sich günstig auf den Erhalt der Linksherzstruktur und -funktion auswirken.

Obstructive sleep apnea syndrome (OSAS) is a common chronic disorder characterized by recurrent episodes of upper respiratory tract obstruction and hypoxia during sleep, leading to the occurrence of loud snoring, breathing interruptions, frequent awakenings or insomnia, morning fatigue, daytime sleepiness, attention deficit, and cognitive dysfunction[18]. The diagnosis and severity of OSAS are usually assessed by the total number of apnea and hypopnea episodes per hour of sleep, namely, the apnea hypopnea index (AHI). This condition is highly prevalent in the general population, affecting 2%–15% of adults [7, 20, 21, 23, 44, 56, 57], particularly those older than 65 years.

Obstructive sleep apnea syndrome is not only associated with a higher risk of occupational accidents but also leads to more cardiovascular diseases such as cardiac remodeling and dysfunction [7, 41]. Echocardiography is a noninvasive, low-cost, time-saving and accurate tool for assessing alterations in cardiac structure and function, and has been widely used in the clinic. To date, there have been some studies exploring the alternations in echocardiographic parameters in OSAS patients. However, large-scale clinical controlled trials on this field are still lacking, and most of the related research had small sample sizes and used inconsistent inclusion and exclusion criteria.

Owing to the overall low awareness of the relationship between OSAS and left ventricular (LV) dysfunction in clinical management, the aim of the current study was to summarize the association between OSAS and LV function and structure, in order to provide guidelines for clinical decision-making.

## Methods

### Data source, search strategy, and selection criteria

Electronic databases including PubMed, Embase, and the Cochrane Library were searched from database inception to March 2018 using the following terms: “ventricular function, left”; “ventricular dysfunction, left”; “heart failure”; “echocardiography”; “sleep apnea, obstructive”; and “sleep-disordered breathing” (see Supplementary Table S1). In addition, we also checked the reference lists of identified reports for other potentially relevant studies. The literature search and study selection were undertaken by two reviewers independently. Any inconsistencies were settled by another researcher. A systematic review was conducted in accordance with the Preferred Reporting Items for Systematic Reviews and Meta-Analyses (PRISMA).

The following criteria were used to identify potentially suitable studies: (1) articles were written fully in English; (2) enrolled participants were all adults (older than 18 years); (3) OSAS was diagnosed by polysomnography and assessed with the AHI; (4) the study included a control group; (5) the study reported at least one of the measures of LV function or structure; (6) there was no significant difference in the body mass index (BMI) of the two groups.

The exclusion criteria were: (1) duplicate reports; (2) studies that had not yet terminated; (3) reviews, case reports, or animal experiments; (4) central sleep apnea; (5) OSAS patients with major comorbidities such as structural heart disease, cardiomyopathy, obstructive or restrictive lung disease demonstrated on pulmonary function testing, pulmonary hypertension, etc.; (6) previous or current treatment for OSAS; (7) studies with hypertension in OSAS group or in control group were excluded in order to avoid the definite effect of hypertension or antihypertension drugs on cardiac remodeling (as an exception, one article was included because the blood pressure level and antihypertensive drugs were both matched between the control group and OSAS group).

### Data extraction and synthesis

Two independent readers screened and extracted data from eligible articles. The following data were extracted from the relevant studies: first author, year of publication, country, number of participants, mean age, BMI, mean AHI, OSAS diagnostic criteria, and LV morphology and function parameters. The main echocardiographic parameters included early diastolic peak flow velocity (E), late diastolic peak flow velocity (A), deceleration time of E wave (DT), early myocardial Doppler peak velocity (E_m),_ late myocardial Doppler peak velocity (A_m_), LV diastolic diameter (LVEDD), LV systolic diameter (LVESD), ejection fraction (EF), isovolumetric relaxation time (IVRT), interventricular septum diameter (IWD), posterior wall diameter (PWD), LV mass (LVM), LV mass index (LVMI), left atrial diameter (LAD), left atrial diameter volume index (LAVI).

### Statistical analysis

Several studies stratified patients on the basis of OSAS severity (mild, moderate, or severe) and reported the grouped LV function data within each stratum. The two formulae presented here were used to combine subgroups and calculate the overall means and standard deviations. However, in the subgroup analysis, each stratum was considered as a separate substudy.

The equations are listed in Supplementary Table S2.

Cochran’s χ^2^ test and the *I* ^2^ statistic were applied to estimate the percentage of variability across studies due to between-study heterogeneity. Heterogeneity was considered statistically significant at *p* < 0.10 and *I* ^2^ > 50%. The weighted mean difference (WMD) with 95% confidence intervals (CIs) was calculated using a fixed-effects model if *I* ^2^ < 50% and using a random-effects model if *I* ^2^ > 50%. Fisher’s *z* test was used to determine the statistical significance of the pooled WMDs. We conducted sensitivity analyses or subgroup analysis if *I* ^2^ > 50%. Egger’s test and Begg’s test were used to examine the presence of publication bias. All analyses were performed using the statistical package Stata, version 14.0 (StataCorp., College Station, TX, USA). The quality of the enrolled studies was evaluated by two independent reviewers according to the Newcastle–Ottawa Scale ranging from 0 to 8.

## Results

### Search results

**Fig. 1:**
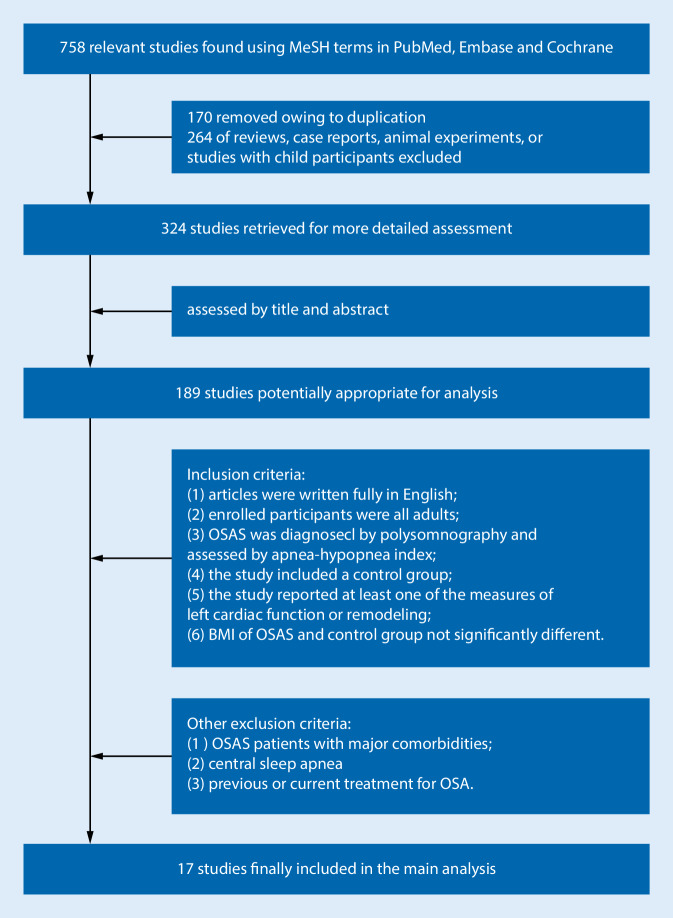
Study selection. *BMI* body mass index, *OSAS* obstructive sleep apnea syndrome

After a strict screening, 739 citations were removed according to the inclusion and exclusion criteria described earlier, and a total of 17 studies with 747 OSAS patients and 426 control participants were included in the final meta-analysis (see Fig. 1).

### Characteristics of studies

The publication years of the eligible studies ranged from 2005 to 2016. The majority of the studies (nine studies, 52.9%) were conducted in Turkey; two studies were conducted in China, two were conducted in Spain, two were conducted in South Korea, one study was conducted in the United States, and one in Italy. In all studies, OSAS was diagnosed and assessed by polysomnography: The control group was defined as having AHI < 5 events/h, while mild, moderate, and severe OSAS were rated as, respectively, 5 ≤ AHI < 15 events/h, 15 ≤ AHI < 30 events/h, and AHI ≥ 30 events/h. For the assessment of left atrial (LA) remodeling, seven studies (41.2%) used LAD and four studies (23.5%) used LAVI. For the assessment of LV remodeling, 13 studies (76.4%) used LVEDD, 11 studies (64.7%) used LVESD, and six studies (35.3%) used LVM. For the assessment of LV function, 15 studies (88.2%) used LVEF. The main characteristics of the studies in this systematic review are listed in Table 1 and the quality of included studies are listed in Supplementary Table S3.

**Table 1:** Characteristics of the included studies

Study	Year of publication	Country	Number of participants	Mean (SD) age (year)	BMI	OSAS diagnostic criteria	Mean (SD) AHI	LA remodeling and dysfunction measures	LV remodeling and dysfunction measures
Kasikcioglu et al. [27]	2005	Turkey	Control (14)	51.8 (12.9)	27.9 (2.5)	PSG: AHI < 5 events/h	1.7 (1.1)	N/A	LVM, LVMI, PWD, IVSd, E, A, E/A, DT, DTc, DT_m_, DT_mc_, E_m_, A_m_, S_m_, E_m_/A_m_
			OSAS (14)	49.7 (11.6)	28.7 (2.9)	PSG: AHI ≥ 15 events/h	32.9 (7.1)		
Ozkececi et al. [37]	2016	Turkey	Control (30)	46.4 (14)	29.3 (4.8)	PSG: AHI ≤ 5 events/h	1 (1–4)	N/A	LVEDD, LVESD, Left Tei index, LVEF
			OSAS (60)	49.6 (11.7)	31.6 (5.8)	PSG: AHI ≥ 5 events/h	24.5 (6–98)		
Altekin et al. [2]	2012	Turkey	Control (21)	45.38 (4.588)	26.35 (4.14)	N/A	N/A	N/A	EF, DVI, SVI, IVSD, PWD, LVM, LAVI, E/A, MPI, DT, E/E′
			Mild (20)	46.95 (6.468)	28.68 (3.44)	5 ≤ AHI < 15 events/h	10.73 (2.57)	N/A	
			Moderate (19)	46.79 (5.029)	29.05 (2.26)	15 ≤ AHI < 30 events/h	20.52 (2.6)	N/A	
			Severe (19)	46.68 (7.660)	29.80 (2.38)	AHI ≥ 30 events/h	58.1 (16.27)	N/A	
Sun et al. [45]	2014	China	Control (50)	62.2 (10.8)	29.66 (4.22)	PSG: AHI < 5 events/h	RDI: 26 (19)	LAD	LVEDD, IVSD, LVPW, LVM, LVMI,
			OSAS (136)	63.3 (10.6)	30.94 (4.15)	PSG: AHI ≥ 5 events/h	RDI: 14 (6)		
Tanriverdi et al. [46]	2006	Turkey	Control (24)	51.9 (5.2)	29.4 (3.9)	PSG: AHI < 5 events/h	3 (1.5)	N/A	IVSD, PWD, LVEDD, LVESD, LVMI, LVEF, peakE/A, E_m_/A_m_
			OSAS (40)	51.3 (9)	29.8 (5.3)	AHI ≥ 5 events/h	25.3 (11.4)	N/A	
Kim et al. [28]	2012	South Korea	Control (24)	48.42 (7.45)	27.45 (2.41)	PSG: AHI < 5 events/h	2.94 (1.44)	LAD、LAVI	IVSD, PWTD, LVMI, RWT, LVEDD, LVESD, LVEF, LVFS
			OSAS (25)	43.48 (11.32)	28.1 (3.1)	AHI ≥ 5 events/h	19.66 (11.64)		
Dursunoglu et al. [17]	2005	Turkey	Control (20)	43.5 (6)	29.3 (2.4)	PSG: AHI < 5 events/h	5.2 (2.8)	LAD	IVSD, PWD, LVEDD, LVESD, E/A, DT, IVRT, LVEF, MPI
			Mild (11)	46.0 (5.6)	30.4 (4.0)	5 ≤ AHI < 15 events/h	25.3 (2.6)		
			Moderate to severe (18)	46.5 (4.9)	30.6 (4)	AHI ≥ 15 events/h	50.1 (11.6)		
Cho et al. [13]	2012	South Korea	Control (20)	47.2 (7.1)	27.9 (1.7)	PSG: AHI < 5 events/h	2.93 (1.44)	LAD	LVEDD, LVESD, IVSTD, PWTD, RWT, LVMI
			OSAS (25)	43.5 (11.3)	28.0 (3.4)	AHI ≥ 5 events/h	19.7 (11.6)		
Varol et al. [50]	2010	Turkey	Control (18)	44.8 (11.6)	29.2 (4.8)	PSG: AHI < 5 events/h	2.1 (1.6)	LAD	IVSD, LVPWD, LVEDD, LVESD, LVM, LVMI, DT, E/A, IVRT, LVEF, MPI
			Mild to moderate (25)	51.2 (8.7)	29.9 (4.3)	5 ≤ AHI ≤ 30 events/h	15.8 (7.4)		
			Severe (21)	48.9 (9.3)	32.2 (3.6)	AHI > 30 events/h	60.7 (24.5)		
Wang et al. [53]	2016	China	Control (30)	45 (6)	25 (4)	PSG: AHI < 5 events/h	2.7 (1.2)	LAV、LAEF、LAVI	LVEDD, LVESD, LVEF, RWTD, LVMI, E, A, E/A, E′, E/E′, DT, IVRT, LVRI
			Mild (26)	48 (8)	26 (4)	5 ≤ AHI < 15 events/h	10.5 (3.2)		
			Moderate (29)	45 (8)	27 (3)	15 ≤ AHI < 30 events/h	18.7 (5.6)		
			Severe (23)	46 (6)	27 (4)	AHI ≥ 30 events/h	57.2 (2.6)		
Vitarelli et al. [51]	2013	Italy	Control (35)	45.1 (12.2)	26.8 (4.3)	PSG: AHI < 5 events/h	3.8 (1.1)	N/A	LVEF, LVMI, IVRT, DT, MPI, E/A
			Mild (19)	48.3 (8.2)	27.5 (5.4)	5 ≤ AHI < 30 events/h	15.4 (2.2)	N/A	
			Severe (23)	47.4 (8.1)	28.3 (6.5)	AHI ≥ 30 events/h	59.4 (9.3)	N/A	
Arias et al. [5]	2005	Spain	Control (15)	48 (9)	28.7 (4.7)	PSG: AHI < 5 events/h	3.9 (3.3)	LAD	E, A, E/A, DT, IVRT, LVESD, LVEDD, LVEF, IVSD, PWD, LVM, LVMI
			OSAS (27)	52 (13)	30.5 (4.0)	AHI ≥ 10 events/h	44.0 (27.5)		
Arias et al. [6]	2006	Spain	Control (10)	50 (10)	27.7 (3)	PSG: AHI < 5 events/h	4.2 (3.5)	LAD	E, A, E/A, DT, LAD, LVEDD, LVESD, LVSF, LVEF, IVSD, LVM, LVMI, LVPWD
			OSAS (23)	51 (13)	30.9 (4)	AHI ≥ 10 events/h	44.1 (29.3)		
Altiparmak et al. [3]	2016	Turkey	Control (35)	43.0 (6.4)	26.2 (3.2)	PSG: AHI < 5 events/h	N/A	LAVI	LVEDD, LVESD, DT, E′, E/A, E/E′, LVMI,
			OSAS (31)	45.5 (6.6)	26.7 (2.1)	AHI ≥ 5 events/h	45.4 (28.1)		
Balci et al. [8]	2012	Turkey	Control (33)	41.6 (11.6)	26.3 (1.4)	PSG: AHI < 5 events/h	3.2 (1.9)	N/A	IVSD, PWD, LVEDD, LVESD, E/A, DT, LVEF, MPI, LVMI, LV MPI
			Mild to moderate (30)	42.5 (11.2)	26.9 (2.4)	5 ≤ AHI < 30 events/h	14.2 (14.6)	N/A	
			Severe (31)	45.7 (10.3)	27.3 (2.3)	AHI ≥ 30 events/h	66.3 (39.9)	N/A	
Tavil et al. [47]	2007	Turkey	Control (29)	49 (11)	29 (5)	PSG: AHI < 5 events/h	2.5 (0.8)	N/A	LVEDD, LVEDD, LVESD, IWSD, PWD, LVEF, LVMI, E, A, E/A, IVRT, E′, A′, E′/A′
			OSAS (29)	48 (10)	29 (6)	AHI ≥ 5 events/h	25 (16)	N/A	
Otto et al. [36]	2007	USA	Control (18)	45 (2)	32.3 (0.9)	PSG: AHI < 5 events/h	2 (0.4)	LAVI	LVMI, EF, DT, IVRT
			OSAS (23)	45 (3)	33.7 (0.8)	AHI ≥ 15 events/h	50 (7)		

*OSAS* obstructive sleep apnea syndrome, *AHI* apnea–hypopnea index, *BMI* body mass index, *E* early diastolic peak flow velocity, *A* late diastolic peak flow velocity, *DT* deceleration time of E wave, *DTc* heart-rate-corrected DT; *DT*_*m*_ Em-wave deceleration time, *DT*_*mc*_ heart-rate-corrected DT_m_, *E*_*m*_ early myocardial Doppler peak velocity, *A*_*m*_ late myocardial Doppler peak velocity, *S*_*m*_ peak velocity of myocardial systolic wave, *LVEDD* left ventricular diastolic diameter, *LVESD* left ventricular systolic diameter, *LVEF* LV ejection fraction, *IVRT* isovolumetric relaxation time, *IWDs* interventricular septum diameter, *PSG* polysomnography, *PWD* posterior wall diameter, *LVM* left ventricular mass, *LVMI* left ventricular mass index, *N/A* not applicable

### Meta-analysis

Table 2 summarizes the data for all the parameters of LA structure, LV structure, and LV function determined by the meta-analysis. Heterogeneity was obvious in the assessment of several parameters, which was speculated as a result of the inconsistent degree of severity of OSAS, varied ages of participants, BMI, geographical location, and duration of OSAS.

**Table 2:** Results of the meta-analysis comparing OSAS patients and controls

Echocardiographic parameters	Number of studies	OSAS/control	WMD (95%CI)	*p*	Study heterogeneity			Egger’s test *p*	Begg’s test *p*
					*I* ^2^ (%)	χ^2^	*p*		
LVEDD (mm)	13	563/319	1.24 (0.68, 1.80)	<0.001	0.0	9.52	0.658	0.431	0.951
LVESD (mm)	11	396/234	1.14 (0.47, 1.81)	0.001	0.0	7.31	0.696	0.722	1.00
LVM	6	304/128	35.34 (20.67, 50.00)	<0.001	79.1	66.05	<0.001	0.914	0.917
LVEF (%)	15	710/394	−3.01 (−1.90, −0.79)	0.001	64.7	39.72	<0.001	0.048	0.038
LAD	7	311/157	2.13 (1.48, 2.77)	<0.001	2.2	6.13	0.408	0.072	0.05
LAVI	3	159/69	3.96 (3.32, 4.61)	<0.001	0.0	1.62	0.445	0.735	1.000

*OSAS* obstructive sleep apnea syndrome, *LVEDD* left ventricular diastolic diameter, *LVESD* left ventricular systolic diameter, *LVM* left ventricular mass, *LVMI* left ventricular mass index, *LVEF* LV ejection fraction, *E* early diastolic peak flow velocity, *A* late diastolic peak flow velocity, *CI* confidence interval

#### Left ventricular structure

The parameters LVEDD, LVESD, and LVM were used to assess LV structure.

**Fig. 2:**
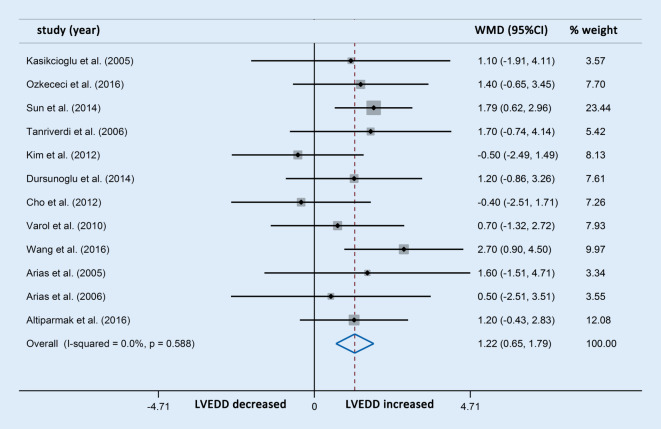
Forest plot of the differences in left ventricular end diastolic diameter (*LVEDD*) between the patients with obstructive sleep apnea syndrome and healthy controls based on echocardiography. *WMD* weighted mean difference

**Fig. 3:**
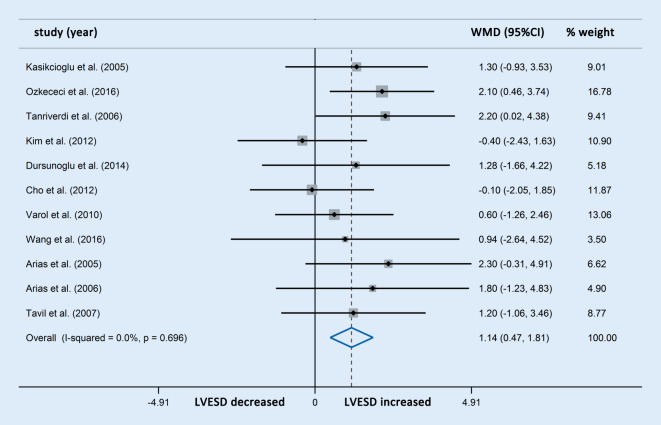
Forest plot of the differences in left ventricular end-systolic diameter (*LVESD*) between the patients with obstructive sleep apnea syndrome and healthy controls based on echocardiography. *WMD* weighted mean difference

Differences in LVEDD were reported in 13 studies involving 563 OSAS patients and 319 control participants. The meta-analysis showed that the LVEDD in patients with OSAS was significantly higher compared with the controls (WMD [95% CIs]: 1.24 [0.68, 1.80]; *p* < 0.001; nonsignificant heterogeneity; Fig. 2). In addition, differences in LVESD were reported in 11 studies involving 396 OSAS patients and 243 control participants, which showed that patients with OSAS also had significantly increased LVESD compared with the controls (WMD [95% CIs]: 1.14 [0.47, 1.81]; *p* = 0.001; nonsignificant heterogeneity; Fig. 3). The subgroup analysis was not performed owing to the limited number of studies with OSAS stratification.

**Fig. 4:**
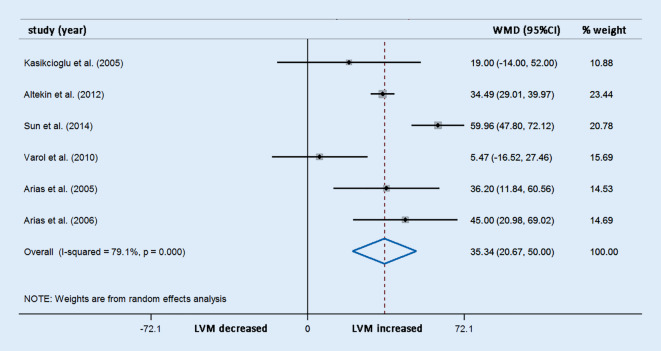
Forest plot of the differences in left ventricular mass (*LVM*) between patients with obstructive sleep apnea syndrome patients and healthy controls based on echocardiography. *WMD* weighted mean difference

**Fig. 5:**
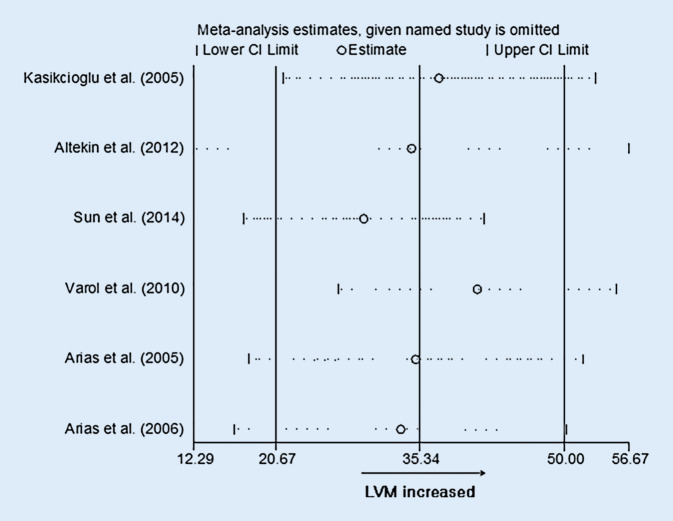
Sensitivity analyses of left ventricular mass (*LVM*). *CI* confidence interval

Differences in LVM were reported in six studies involving 304 OSAS patients and 128 control participants. The meta-analysis demonstrated that the LVM (WMD [95% CIs]: 35.34 [20.67, 50.00]; *p* < 0.001; Fig. 4) was significantly elevated compared with the controls. However, potential evidence of significant heterogeneity was detected, and thus sensitivity analysis was conducted. After exclusion of each study from the pooled analysis, the WMDs were always >0, which confirmed the reliability of these results (Fig. 5).

#### Left ventricular function

**Fig. 6:**
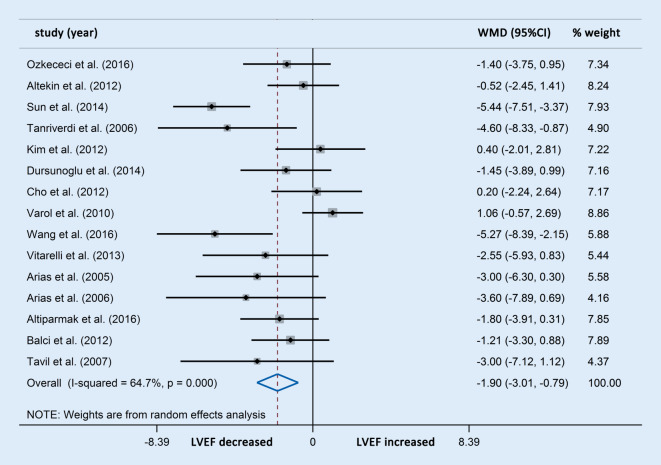
Forest plot of the differences in left ventricular ejection fraction (*LVEF*) between patients with obstructive sleep apnea syndrome and healthy controls based on echocardiography. *WMD* weighted mean difference

Left ventricular ejection fraction was used to assess LV systolic function and LV diastolic function. Differences in LVEF were reported in 15 studies involving 710 OSAS patients and 394 control participants. The LVEF in patients with OSAS decreased significantly compared with the controls (WMD [95% CIs]: −3.01 [−1.90, −0.79]; *p* < 0.001; Fig. 6).

**Fig. 7:**
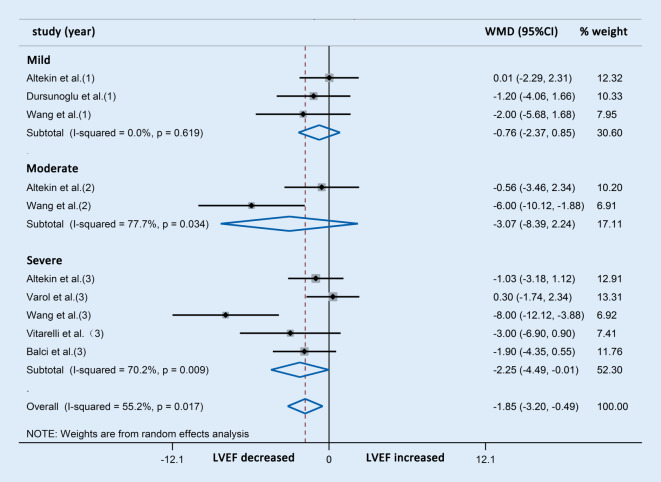
Forest plot of the differences in left ventricular ejection fraction (*LVEF*) between patients with obstructive sleep apnea syndrome and healthy controls based on echocardiography (subgroup analysis). *WMD* weighted mean difference

Further subgroup analysis indicated that the reduction in LVEF was consistent with the severity of OSAS (Fig. 7).

**Fig. 8:**
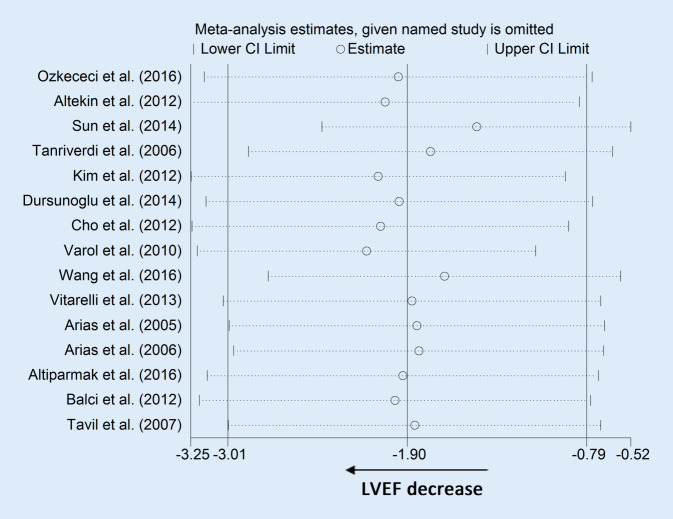
Sensitivity analyses of left ventricular ejection fraction (*LVEF*). *CI* confidence interval

Significant heterogeneity was detected in the analysis of LVEF. However, sensitivity analysis showed that after exclusion of each study from the pooled analysis, the results were reliable (Fig. 8).

#### Left atrial structure

The parameters LAD and LAV were used to evaluate LA structure.

**Fig. 9:**
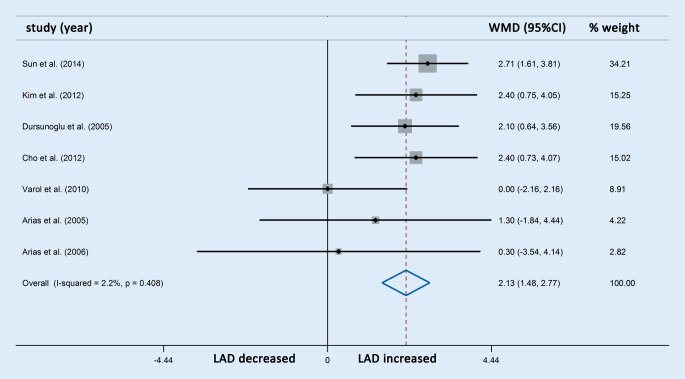
Forest plot of the differences in left atrial diameter (*LAD*) between patients with obstructive sleep apnea syndrome and healthy controls based on echocardiography. *WMD* weighted mean difference

**Fig. 10:**
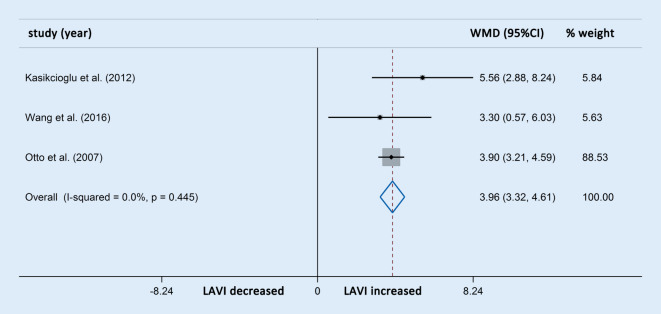
Forest plot of the differences in left atrial diameter volume index (*LAVI*) between patients obstructive sleep apnea syndrome and healthy controls based on echocardiography. *WMD* weighted mean difference

Differences in LAD were reported in seven studies involving 311 OSAS patients and 157 control participants. The meta-analysis showed that the LAD in patients with OSAS was significantly higher than in the control group (WMD [95% CIs]: 2.13 [1.48, 2.77]; *p* < 0.001; nonsignificant heterogeneity; Fig. 9). The LAVI was also compared through an analysis of four studies comprising 184 OSAS patients and 93 control participants. Owing to apparent heterogeneity, sensitivity analysis was conducted and one study was accordingly removed. The three studies finally selected involving 159 OSAS patients and 69 control participants showed that patients with OSAS also had significantly increased LAVI compared with the controls (WMD [95% CIs]: 3.96 [3.32, 4.61]; *p* < 0.001; nonsignificant heterogeneity; Fig. 10). Neither subgroup analysis of the LAD nor of the LAVI was performed because of the limited number of studies with OSAS stratification.

## Discussion

### Main findings

To our knowledge, this is the first meta-analysis to systematically evaluate changes in left cardiac structure and function in OSAS patients. Particularly, the effect of systemic hypertension was eliminated in the current analysis by excluding those studies with hypertensive patients enrolled, since many ultrasonic parameters may be affected by hypertension. Thus, we demonstrate that OSAS induces cardiac abnormality independent of systemic hypertension. Finally, 17 case–control studies were included in this meta-analysis with a total of 747 patients with OSAS and 426 controls.

We found that alterations in the echocardiographic parameters of LV and LA remodeling, including an increase in LVEDD, LVESD, LVM, LAD, and LAVI, were important features in OSA patients without major comorbidities, which indicated that OSAS resulted in enlargement and hypertrophy of the left ventricle and atrium. Moreover, significant decreases in LVEF were observed in OSAS patients. It should be noted that the alterations in LVEF seemed not to be remarkable enough to induce obvious clinical symptoms of LV dysfunction.

In the analysis of LVEF, we found significant heterogeneity between studies. The heterogeneity is to be expected given the variation in disease severity. However, subgroup analysis still showed significant heterogeneity, which may stem from the variety of studies conducted by different teams at various geographic locations as well as the differences in the patient populations.

### LV remodeling and dysfunction in OSAS patients

It has been reported that intermittent hypoxia could induce LV remodeling, which was regarded as the basis of LV dysfunction caused by OSAS [15]. Although the negative effect of intermittent hypoxia on the cardiovascular system has not been completely clarified, multiple putative mechanisms including sympathetic overactivation, oxidative stress, inflammation, metabolic deregulation, and endothelial dysfunction have been suggested to be involved.

Hypoxemia, hypercapnia, and acidosis, induced by chronic intermittent hypoxia in OSAS patients, could activate the sympathetic nervous system. Prior studies showed that patients with metabolic syndrome and comorbid OSAS have a higher sympathetic drive than do patients with metabolic syndrome without OSAS [22, 49]. Furthermore, Scala et al. reported a significant correlation between OSAS and cardiac adrenergic impairment in patients with heart failure [43]. The exposure to intermittent hypoxia and hypercapnia, elicited by OSAS, increases muscle sympathetic nerve activity (MSNA) acutely during sleep [10], and the over-activation of the sympathetic nervous system is persistent even after removal of the hypoxic stimulus [4, 54]. Bradley et al. reported that LV transmural pressure was increased in response to the generation of negative intrathoracic pressure (Pit) and the elevation of systemic blood pressure secondary to hypoxia-induced sympathetic nervous system activation [11]. This results in the reduction of LV preload and increased afterload, which can directly affect LV systolic function [26]. The combination of increased LF afterload and increased heart rate (HR) promotes myocardial oxygen demand, thus brings a higher risk of cardiac ischemia and arrhythmias, and chronically contributes to LV hypertrophy and failure. It is well known that the sympathetic nervous system is the most prominent among neurohormonal mechanisms in HF, which pushes the heart to work at an overloaded level, and confers significant toxicity to the failing heart and increases morbidity and mortality in chronic decompensated HF [32].

In addition, systemic inflammation triggered by OSAS plays an important role in intermittent hypoxia-induced LV remodeling, as was confirmed by elevated plasma levels of tumor necrosis factor-alpha (TNF-α) and interleukin‑6 (IL-6) in patients with OSA [34]. Inflammatory response and free radical generation could cause an imbalance between myocardial oxidation and antioxidant activity, result in myocardial injury, and increase susceptibility to myocardial ischemia [19]. The consequent myocardial ischemia leads to a shortage in ATP production, inorganic phosphate accumulation, and myocardial acidosis, which inhibit excitement–contraction coupling and cause regional ventricular systolic dysfunction [40]. Besides, in the case of OSAS, exposure to intermittent hypoxia elevates blood pressure, and thus may induce functional, mechanical, and structural changes in the aortic wall in response to hemodynamic and biomechanical stress, which has been verified in mice [12]. Hypoxia stimulates the elastic fiber network with disorganization, fragmentation, and estrangement between the endpoints of disrupted fibers, and induces collagen and mucoid interlaminar accumulation in the extracellular matrix as well as LF perivascular fibrosis. These cardiovascular remodeling events induced by hypoxia were normalized after continuous positive airway pressure (CPAP) treatment.

The hemodynamic changes associated with obesity might contribute to the increased LV mass observed in patients with OSA. Of interest, Parisi et al. have pointed out that HF patients with sleep disordered-breathing have higher epicardial adipose tissue (EAT) thickness values than do HF patients without nocturnal apneas [39], which indicates EAT might be a pathophysiological link between OSAS and increased cardiovascular risk [31, 33]. Besides, CPAP therapy reduces EAT and significantly ameliorates cardiometabolic parameters in obese OSAS patients [29]. Moreover, EAT is a source of several adipocytokines and directly affects the myocardium through vasocrine and paracrine mechanisms [24]. Exposure to hypoxia leads to higher production of factor-1a and Fos-like antigen (FOSL) 2, resulting in up-regulation of leptin expression in the EAT, along with increased vascularization, inflammation, and fibrosis [16]. It has been demonstrated that EAT thickness has a high correlation with sympathetic nervous system hyperactivity and it is a local source of catecholamines in HF patients, which suggests sympathetic nervous system hyperactivity is a potential pathophysiological link between sleep apneas and EAT in HF [38].

Overall, increased sympathetic activity, endothelial dysfunction, systemic inflammation, oxidative stress, and metabolic anomalies induced by intermittent hypoxia play major roles in the progression of LV remodeling and dysfunction.

### LA remodeling in OSAS patients

Our analysis revealed a significant increase in LA volume in patients with OSAS. Left atrial enlargement was considered to occur owing to elevated LV pressure and diastolic dysfunction. Sympathetic activation, decreased parasympathetic tone, and inflammation associated with intermittent hypoxia might contribute to atrial structural and electrical remodeling.

It should be noted that recent evidence has indicated OSAS patients might be predisposed to the development and recurrence of atrial fibrillation [48]. The forced inspiration-induced acute LA dilation related to diastolic dysfunction may be an important component of the arrhythmogenic substrate for atrial fibrillation during sleep apnea episodes [9, 25]. More explorations of treatment for OSAS, such as CPAP, are expected to alter the anticipated frequency of OSAS-related cardiac arrhythmias.

### RV remodeling and dysfunction in OSAS patients

The correlation between OSAS and right ventricular (RV) remodeling and dysfunction has also raised intense interest from researchers, but the conclusions from various have differed. Yang et al. found the inner diameters and the anterior wall thickness of the RV were increased in patients with severe OSAS and deteriorated as the disease course progressed [55]. Vitarelli et al. reported that RV dilatation and dysfunction were significantly associated with AHI and the severity and frequency of apnea episodes [52]. Similarly, several other studies reported that OSAS patients presented with right cardiac dysfunction [1, 30]. By contrast, other studies did not reveal any significant changes in RV morphology and function in OSA patients [14, 17].

Several mechanisms were raised to illustrate the potential relationship between OSAS and RV changes. Prior studies suggested that permanent pulmonary hypertension was an important factor. Repetitive nocturnal arterial oxygen desaturation and hypercapnia lead to pulmonary vascular endothelium remodeling and vasoconstriction, thus increasing pulmonary artery pressure [42], leading to RV overload and inducing the release of inflammatory factors. As a result, the compensatory hypertrophic RV gradually presents with dysfunction. However, recent studies pointed out that RV alternations occurred in early stage of OSAS in the absence of pulmonary hypertension [30, 52], indicating that pulmonary hypertension was not the only cause. Some other studies reported the intrathoracic negative pressure against an occluded airway increased venous return and volume overload, and consequently expanded and remodeled the RV [1]. Another possible mechanism is that intermittent hypoxia and CO_2_ retention stimulated central and peripheral chemoreceptors and increased sympathetic nerve activity, which induced RV dysfunction. In addition, LV dysfunction induced by OSAS may also lead to RV dysfunction, since there is a close anatomical association between the two ventricles [35].

Since the effects of OSAS on the LV and RV may be different, we should conduct further analyses to uncover the precise correlation and mechanism between OSAS and RV remodeling and dysfunction.

### Limitations

The present study was a clinical observational study. The follow-up time was not controlled. In our opinion, the duration of OSAS might be a relevant factor when assessing the effect of OSAS on cardiac structure and function, but it is very difficult to assess the duration of OSAS owing the rate of missed diagnoses in the clinic. Besides, this analysis involved no outcome data such as survival rate and risk of cardiovascular events during follow-up. Further research is needed to clarify the effect of OSAS on the long-term prognosis of patients. Additional analyses may identify the correlation and precise mechanisms between OSAS and RV remodeling.

## Conclusion

On the basis of this meta-analysis, we conclude that patients with obstructive sleep apnea syndrome (OSAS) display increased left atrial dilatation and left ventricular (LV) hypertrophy and dilatation, along with impaired LV systolic function. Therefore, more emphasis should be placed on the clinical significance of OSAS in cardiovascular risk. Treatment against OSAS such as continuous positive airway pressure can attenuate sleep apnea and might prevent the decrease in LV ejection fraction in the long term.

## Caption Electronic Supplementary Material

Supplementary Table S1. Search Strategy.

Supplementary Table S2. Equation.

Supplementary Table S3. Quality of included studies.
