# Supplementary material for: Left ventricular remodeling and dysfunction in obstructive sleep apnea: Systematic review and meta-analysis
Source: Herz. 2019 Sep 25;45(8):726–38. doi: 10.1007/s00059-019-04850-w (PMC7695673; doi:10.1007/s00059-019-04850-w)
Supplement: Supplementary file 1 — Supplementary Table S1. Search Strategy. [file 59_2019_4850_MOESM1_ESM.docx]

**Supplementary Table S1: Search Strategy**

| Pubmed | Search Strategy |
| --- | --- |
| ＃1 | (Ventricular Function, Left) Mesh OR (Left Ventricular Function) OR (Function, Left Ventricular) OR (Functions, Left Ventricular) OR (Left Ventricular Functions) OR (Ventricular Functions, Left) |
| ＃2 | (Ventricular Dysfunction, Left) Mesh (Left Ventricular Dysfunction) OR (Dysfunction, Left Ventricular) OR (Dysfunctions, Left Ventricular) OR (Left Ventricular Dysfunctions) OR (Ventricular Dysfunctions, Left) |
| ＃3 | (Heart Failure) Mesh (Cardiac Failure) OR (Heart Decompensation, Heart) OR (Heart Failure, Right-Sided) OR (Heart Failure, Right Sided) OR (Right-Sided Heart Failure) OR (Right Sided Heart Failure) OR (Myocardial Failure) OR (Congestive Heart Failure) OR (Heart Failure, Congestive) OR (Heart Failure, Left-Sided) OR (Heart Failure, Left Sided) OR (Left-Sided Heart Failure) OR (Left Sided Heart Failure) |
| ＃4 | ＃1 or ＃2 or ＃3 308626 |
| ＃5 | (Echocardiography) Mesh OR (Transthoracic Echocardiography) OR (Echocardiography, Transthoracic) OR (Echocardiography, Cross-Sectional) OR (Echocardiography, Cross Sectional) OR (Cross-Sectional Echocardiography) OR (Cross Sectional Echocardiography) OR (Echocardiography, M-Mode) OR (Echocardiography, M Mode) OR (M-Mode Echocardiography) OR (M Mode Echocardiography) OR (Echocardiography, Contrast) OR (Contrast Echocardiography) OR (2D Echocardiography) OR (Echocardiography, Two-Dimensional) OR (Echocardiography, Two Dimensional) OR (Echocardiography, 2D) OR (Echocardiography, 2-D) OR (Echocardiography, 2 D) OR (Two-Dimensional Echocardiography) OR (Two Dimensional Echocardiography) OR (2-D Echocardiography) OR (2 D Echocardiography) |
| ＃6 | ＃4 and ＃5 54823 |
| ＃7 | (Sleep Apnea, Obstructive)[Mesh] OR (Apneas, Obstructive Sleep) OR (Obstructive Sleep Apneas) OR (Sleep Apneas, Obstructive) OR (Obstructive Sleep Apnea Syndrome) OR (Obstructive Sleep Apnea) OR (OSAHS) OR (Syndrome, Sleep Apnea, Obstructive) OR (Sleep Apnea Syndrome, Obstructive) OR (Apnea, Obstructive Sleep) OR (Sleep Apnea Hypopnea Syndrome) OR (Syndrome, Obstructive Sleep Apnea) OR (Upper Airway Resistance Sleep Apnea Syndrome) OR (Syndrome, Upper Airway Resistance, Sleep Apnea) |
| ＃8 | (sleep-disordered breathing)[Mesh] OR (Apnea Syndrome, Sleep) OR (Apnea Syndromes, Sleep) OR (Sleep Apnea Syndrome) OR (Apnea, Sleep) OR (Apneas, Sleep) OR (Sleep Apnea) OR (Sleep Apneas) OR (Sleep Hypopnea) OR (Hypopnea, Sleep) OR (Hypopneas, Sleep) OR (Sleep Hypopneas) OR (Sleep-Disordered Breathing) OR (Breathing, Sleep-Disordered) OR (Sleep Disordered Breathing) OR (Sleep Apnea, Mixed Central and Obstructive) OR (Mixed Central and Obstructive Sleep Apnea) OR (Sleep Apnea, Mixed) OR (Mixed Sleep Apnea) OR (Mixed Sleep Apneas) OR (Sleep Apneas, Mixed) OR (Hypersomnia with Periodic Respiration) |
| ＃9 | ＃7 or ＃8 41618 |
| ＃10 | ＃6 and ＃9 337 |
