# Supplementary figures and images for: Left ventricular remodeling and dysfunction in obstructive sleep apnea: Systematic review and meta-analysis
Source: Herz. 2019 Sep 25;45(8):726–38. doi: 10.1007/s00059-019-04850-w (PMC7695673; doi:10.1007/s00059-019-04850-w)

**Supplementary Table S2. Equation**


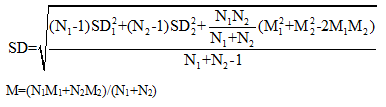


M=(N1M1+N2M2)/(N1+N2)

Supplement: Supplementary file 2 — Supplementary Table S2. Equation. [file 59_2019_4850_MOESM2_ESM.docx]
