# Supplementary material for: Left ventricular remodeling and dysfunction in obstructive sleep apnea: Systematic review and meta-analysis
Source: Herz. 2019 Sep 25;45(8):726–38. doi: 10.1007/s00059-019-04850-w (PMC7695673; doi:10.1007/s00059-019-04850-w)
Supplement: Supplementary file 3 — Supplementary Table S3. Quality of included studies. [file 59_2019_4850_MOESM3_ESM.docx]

**Supplementary Table S3. Quality of included studies**

| Study | Year of publication | Selection | | | | | Comparability | Exposure | | Total scores |
| --- | --- | --- | --- | --- | --- | --- | --- | --- | --- | --- |
|  |  | Is the case definition adequate? | Representativeness of the case | Selection of controls | Definition of`controls | Comparability of cases and controls on the basis of the Design or Analysis | Ascertainment exposure | Same method of ascertainment for cases and controls | Non-Response rate |  |
| Kasikcioglu et al.^[^[^10^](#_ENREF_10)^]^ | 2005 | 1 | 0 | 1 | 1 | 2 | 1 | 1 | 0 | 7 |
| Ozkececi et al.^[^[^11^](#_ENREF_11)^]^ | 2016 | 1 | 0 | 1 | 1 | 2 | 1 | 1 | 0 | 7 |
| Altekin et al.^[^[^12^](#_ENREF_12)^]^ | 2012 | 1 | 0 | 1 | 1 | 1 | 1 | 1 | 0 | 6 |
| Sun et al.^[^[^13^](#_ENREF_13)^]^ | 2014 | 1 | 0 | 1 | 1 | 1 | 1 | 1 | 0 | 6 |
| Tanriverdi et al.^[^[^14^](#_ENREF_14)^]^ | 2006 | 1 | 0 | 1 | 1 | 1 | 1 | 1 | 0 | 6 |
| Vural et al.^[^[^15^](#_ENREF_15)^]^ | 2014 | 1 | 0 | 1 | 1 | 2 | 1 | 1 | 0 | 7 |
| Kim et al.^[^[^16^](#_ENREF_16)^]^ | 2012 | 1 | 0 | 1 | 1 | 2 | 1 | 1 | 0 | 7 |
| Dursunoglu et al.^[^[^17^](#_ENREF_17)^]^ | 2005 | 1 | 0 | 1 | 1 | 2 | 1 | 1 | 0 | 7 |
| Cho et al.^[^[^18^](#_ENREF_18)^]^ | 2012 | 1 | 0 | 1 | 1 | 1 | 1 | 1 | 0 | 6 |
| Varol et al.^[^[^19^](#_ENREF_19)^]^ | 2010 | 1 | 0 | 1 | 1 | 2 | 1 | 1 | 0 | 7 |
| Butt et al.^[^[^20^](#_ENREF_20)^]^ | 2012 | 1 | 1 | 1 | 1 | 1 | 1 | 1 | 0 | 7 |
| Wang et al.^[^[^21^](#_ENREF_21)^]^ | 2016 | 1 | 1 | 1 | 1 | 2 | 1 | 1 | 0 | 8 |
| Vitarelli et al.^[^[^22^](#_ENREF_22)^]^ | 2013 | 1 | 0 | 1 | 1 | 2 | 1 | 1 | 0 | 7 |
| Arias et al.^[^[^23^](#_ENREF_23)^]^ | 2005 | 1 | 1 | 1 | 1 | 2 | 1 | 1 | 0 | 8 |
| Arias et al.^[^[^24^](#_ENREF_24)^]^ | 2006 | 1 | 0 | 1 | 1 | 2 | 1 | 1 | 0 | 7 |
| Altiparmak et al.^[^[^25^](#_ENREF_25)^]^ | 2016 | 1 | 1 | 1 | 1 | 2 | 1 | 1 | 0 | 8 |
| Balci et al.^[^[^26^](#_ENREF_26)^]^ | 2012 | 1 | 0 | 1 | 1 | 2 | 1 | 1 | 0 | 7 |
| Tavil et al.^[^[^27^](#_ENREF_27)^]^ | 2007 | 1 | 0 | 1 | 1 | 2 | 1 | 1 | 0 | 7 |
| Otto et al.^[^[^28^](#_ENREF_28)^]^ | 2007 | 1 | 0 | 1 | 1 | 2 | 1 | 1 | 0 | 7 |
